# Patterns of differential gene expression in adult rotation-resistant and wild-type western corn rootworm digestive tracts

**DOI:** 10.1111/eva.12278

**Published:** 2015-07-16

**Authors:** Chia-Ching Chu, Jorge A Zavala, Joseph L Spencer, Matías J Curzi, Christopher J Fields, Jenny Drnevich, Blair D Siegfried, Manfredo J Seufferheld

**Affiliations:** 1 Department of Crop Sciences, University of IllinoisUrbana, IL, USA; 2 Facultad de Agronomía, Cátedra de Bioquímica INBA-CONICET, University of Buenos Aires-CONICETBuenos Aires, Argentina; 3 Illinois Natural History Survey, University of IllinoisChampaign, IL, USA; 4 DuPont PioneerSalto, Buenos Aires, Argentina; 5 High-Performance Biological Computing, Roy J. Carver Biotechnology CenterUrbana, IL, USA; 6 Department of Entomology, University of NebraskaLincoln, NE, USA; 7 Department of Entomology, University of IllinoisUrbana, IL, USA

**Keywords:** adaptation, agriculture, ecological genetics

## Abstract

The western corn rootworm (WCR,*Diabrotica virgifera virgifera* LeConte) is an important pest of corn. Annual crop rotation between corn and soybean disrupts the corn-dependent WCR life cycle and is widely adopted to manage this pest. This strategy selected for rotation-resistant (RR) WCR with reduced ovipositional fidelity to corn. Previous studies revealed that RR-WCR adults exhibit greater tolerance of soybean diets, different gut physiology, and host–microbe interactions compared to rotation-susceptible wild types (WT). To identify the genetic mechanisms underlying these phenotypic changes, a *de novo* assembly of the WCR adult gut transcriptome was constructed and used for RNA-sequencing analyses of RNA libraries from different WCR phenotypes fed with corn or soybean diets. Global gene expression profiles of WT- and RR-WCR were similar when feeding on corn diets, but different when feeding on soybean. Using network-based methods, we identified gene modules transcriptionally correlated with the RR phenotype. Gene ontology enrichment analyses indicated that the functions of these modules were related to metabolic processes, immune responses, biological adhesion, and other functions/processes that appear to correlate to documented traits in RR populations. These results suggest that gut transcriptomic divergence correlated with brief soybean feeding and other physiological traits may exist between RR- and WT-WCR adults.

## Introduction

The western corn rootworm (WCR, *Diabrotica virgifera virgifera* LeConte; Coleoptera: Chrysomelidae) is an important corn pest in the US and Europe (Gray et al. 2009). Its subterranean larvae mainly feed on corn roots, while adult beetles feed on aboveground corn tissues (Levine and Oloumi-Sadeghi 1991). Female WCR often mate soon after emergence (Hill 1975) and eventually oviposit in the soil of nearby cornfields (Levine and Oloumi-Sadeghi 1991; Spencer et al. 2009). These characteristics made annual crop rotation between corn and nonhost soybeans (*Glycine max*) an effective measure to control the pest; larvae emerging in soybean fields (planted after corn) do not survive (Spencer et al. 2009). However, within a few decades, the selection pressure imposed by wide adoption of crop rotation resulted in the appearance and spread of a rotation-resistant (RR) WCR variant in the eastern portion of the US Corn Belt (Levine et al. 2002; Gray et al. 2009). It has been suggested that RR-WCR were selected for lower ovipositional fidelity to cornfields and higher mobility (Levine et al. 2002; Knolhoff et al. 2006), both of which may help them to circumvent crop rotation. In areas where rotation resistance is a problem, WCR could often be found feeding and ovipositing in both soybean and cornfields (Levine et al. 2002; Spencer et al. 2009).

Soybeans are well-defended against many Coleopteran insects by cysteine protease inhibitors (CystPIs). Previous studies showed that ingestion of CystPIs could inhibit WCR digestive proteolysis in laboratory and field conditions (Zhao et al. 1996; Koiwa et al. 2000; Zavala et al. 2008). Adult WCR fed with CystPI also exhibit reduced fecundity (Kim and Mullin 2003). A recent study across different WT- and RR-WCR populations indicated that RR populations exhibit higher tolerance of soybean, greater gut cysteine protease activity, and elevated protease gene expression compared to WT populations (Curzi et al. 2012). One of the WT populations collected from Ames, Iowa, a location closer to the western edge of the reported rotation-resistance problem area (Gray et al. 2009) (Figure S1), exhibited digestive cysteine protease activity and tolerance of soybean diet that were slightly higher than those of other WT populations (Curzi et al. 2012). This suggests that low levels of RR could have existed in Ames, Iowa. In another study, the gut bacterial community structure of RR-WCR populations was found to differ from that of WT populations; antibiotic experiments also indicated that the gut microbiota contribute to RR-WCR’s elevated tolerance to soybean CystPIs (Chu et al. 2013).

Although the protease activity and the gut microbiota of RR-WCR may act as important components helping RR-WCR to confront dietary stress, their actions may be accompanied by alterations in RR-WCR genetics at a broader scale. Insect physiology, behavior and host–microbe interactions are interconnected and genetically regulated and are vital for determining their fitness in surrounding environments (Simpson and Raubenheimer 1993; Dillon and Dillon 2004; Lee and Park 2004; Schmid-Hempel 2005). Characterizing gene regulatory changes in RR-WCR may open venues for developing tools for monitoring their spread and presence in the field and broaden our understanding of resistance evolution. Here, we propose that the RR-WCR’s adaptation to brief soybean herbivory is not only correlated with changes in few aspects of their gut physiology, but is also influenced by substantial changes in their genetic regulation.

To determine whether transcriptomic divergence exists between WT- and RR-WCR adults, we sampled field populations of WT- or RR-WCR across four states in the USA and studied whether gut transcriptomes of RR-WCR differ from those of WT populations. Also, the overall relationships between these transcriptomes and those from WCR collected from Ames, Iowa – a population that exhibited slightly elevated tolerance of soybean diet – were also examined. A *de novo*assembly of the WCR adult gut transcriptome was constructed and used for RNA-sequencing (RNA-seq) analyses on RNA libraries from different WCR phenotypes that were fed on corn or soybean diets. In addition, differential gene expression analyses, network-based methods (weighted gene co-expression network analysis), and gene ontology (GO) enrichment analysis were used to characterize gene modules transcriptionally correlated with the RR phenotype. The experiments presented in this study examine the potential genetic changes in RR-WCR and suggest that transcriptomic divergence exists between RR and WT populations.

## Materials and methods

### Insect and plant materials

To study the gut transcriptomes of WCR adults, we obtained total gut RNA from field-sampled and laboratory-reared WCR that were fed with corn or soybean tissues. We used the samples for *de novo*assembly of a reference transcriptome (pooling RNA from all population × diet samples) and RNA-seq analyses (pooled within each phenotype × diet) comparing transcription profiles in different WCR phenotypes on different diets (details described in following sections). Adults of six field WCR populations were sampled from different cornfields during late July to August 2010 (Figure S1, Table S1). Three RR-WCR populations were collected from Shabbona (41°50′36″N, 88°50′58″W), Minonk (40°51′26″N, 89°00′26″W), and Urbana (40°09′14″N, 88°08′40″W), Illinois. Two WT-WCR populations were sampled from Higginsville, Missouri (39°07′09″N, 93°49′42″W), and Concord, Nebraska (42°23′39″N, 96°57′23″W). A WT population that exhibited slightly higher tolerance of soybean diet than other WT population in a previous study (Curzi et al. 2012) was collected from Ames, Iowa (42°3′8″N, 93°32′6″W). The populations were grouped into different phenotypes based on the previously documented range of RR-WCR (Gray et al. 2009) and phenotypic characterizations on these populations (Curzi et al. 2012; Chu et al. 2013). The sex ratios for all populations collected were similar (∼70% females). Individuals from a nondiapausing USDA-reared WCR colony were also obtained during 2009; their RNA was used only for construction of the reference transcriptome. Sampled beetles were kept on a diet of immature corn ears and a mixture of green and yellow silks (*Zea mays*, variety ‘Sugar Buns’) in the laboratory for at least 72 h before experiments. Fresh corn diet was provided daily. Corn plants used in this study were grown in an experimental plot on the University of Illinois campus in Urbana-Champaign. Soybean plants (*G. max*‘Williams 82’) were grown in a University of Illinois greenhouse under light intensities of 1200–1500 *μ*mol m^−2^ s^−1^ for 4 weeks.

### Dietary treatments and WCR gut RNA extraction

Caged WCR adults from each population/colony were sampled after exposure to three dietary treatments: (i) the same corn diet described above (designated as ‘0 h’) or a soybean diet (intact soybean plant) for (ii) 8 h or (iii) 36 h. To promote soybean feeding among the soybean treatment groups, insects were starved for 48 h (with water access) before they were given access to soybean plants. All cages were kept in a growth chamber set at 24°C, 70–90% relative humidity and on a 14:10 (L:D) photoperiod. After the treatments, insects were anesthetized using chloroform. Complete digestive tracts were detached from WCR heads and pulled out of the bodies from the posterior end using forceps. The guts were then immediately soaked in 50 *μ*L of RNAlater (Ambion Inc., Austin, TX, USA) and stored (within the solution) at −80°C until use. For RNA extraction, gut samples (each sample consisted of complete digestive tracts from five beetles) were homogenized in liquid nitrogen using mortars and pestles. Total RNA was extracted from these samples using the E.Z.N.A. Total RNA Kit I (Omega Bio-Tek, Norcross, GA, USA) including DNase treatments. A total of 210 insects were used in this study.

### *De novo*assembly of the WCR adult gut transcriptome

To construct a reference transcriptome, gut RNA was collected from seven WCR populations subjected to the three dietary treatments described above. Two biological replicates from each ‘population × diet’ treatment were sampled. A total of 42 RNA samples (7 populations × 3 dietary treatments × 2 biological replicates) were pooled at equal mass (to 21 *μ*g) into a ‘454 sequencing sample’. The sample was 454 sequenced using standard protocols (Appendix S1). The sequencing produced 1.22 million reads (406 mb) with an average read length of ∼333 bp. The data were assembled using Newbler v. 2.7 (Roche Inc., Indianapolis, IN, USA) using an adjusted minimum overlap identity of 95%. In addition, paired-end Illumina reads obtained from the RNA-seq analysis (described below) were incorporated to error-correct homopolymers in the initial 454 assembly using iCORN v. 1.0 (Otto et al. 2010). Transcriptome annotation was conducted using BLASTx v. 2.2.26+ (Altschul et al. 1997; Cameron et al. 2004) against the following databases: UniRef90 (Sept. 2012), *Drosophila melanogaster*(FlyBase release 5.47), *Tribolium castaneum*(BeetleBase OGS3), and the *Arthropoda*subset of the nonredundant (nr) protein database (Sept. 12, 2012; E-value cutoff of 10^−5^). Based on the search results, isogroups (i.e., groups of contigs that correspond to a gene) were then resolved to single best representative coding isotigs/contigs (i.e., mRNA) based on consistency of BLASTX top hits; each isotig/contig has a single, longest best scoring hit to the databases (Figure S2). In several cases, isogroups were split into subgroups if BLAST results indicated isotig sequences may result from two genic regions; each subgroup was then resolved as described above for normal isogroups. Sequences that do not have a protein hit in the above BLAST analysis were mapped (via BLASTN) to a previously annotated WCR larval gut transcriptome (Eyun et al. 2014). These mapped sequences were assumed to be noncoding WCR RNA.

### Illumina RNA sequencing

To cover the transcription profiles across distinct WCR phenotypes fed on different dietary treatments (greater breadth) under reasonable expense, the six previously characterized WCR populations were grouped into different ‘phenotypic’ groups. The Higginsville (Missouri) and Concord (Nebraska) populations were grouped as ‘WT’ populations, whereas the Urbana, Minonk, and Shabbona populations from Illinois were classified as ‘RR’ populations. WCR collected from Ames, Iowa, were sequenced separately (designated as ‘IA’) due to their slightly elevated tolerance of soybean diet (Curzi et al. 2012). For each dietary treatment, RNA samples from populations within each phenotypic group were pooled (at equal mass; resulting in an ‘Illumina sequencing sample’). For example, one RNA sample from Higginsville WCR fed with corn diets was pooled with another from Concord WCR fed with the same corn diet, resulting an independent ‘WT × corn diet’ sample. Two of such sequencing samples (biological replicates) from nine ‘phenotype × diet’ treatments (18 in total) were sequenced using an Illumina HiSeq2000 (Illumina Inc., San Diego, CA, USA; Appendix S1). The single-end sequencing produced 16.8–22.2 million reads (100 bp reads) per library, while the paired-end sequencing produced 16.6–23 million paired reads (forward and reverse reads) per library.

### Illumina data analyses

Reads were mapped using the assembled and annotated nonredundant WCR gut transcriptome using Bowtie 2.0.2 (Langmead and Salzberg 2012). The output SAM (Sequence Alignment/Map) file was parsed to find unique alignments and then converted to BAM format (binary version of SAM) and sorted using SAMtools 0.1.18 (Li et al. 2009). Only uniquely mapping reads were counted, and only tags without ambiguous nucleotides were kept for further analyses. Data analysis was conducted on the combined single end and R1 (forward) of the paired-end reads using R 2.15.2 (R Core Team 2013) and packages noted below. After quality assessment, 1123 isotigs with unusually consistent expression patterns (potential artifacts of the alignment/count process) were removed from the following analysis. Additionally, 590 isotigs were removed prior to analyses because they did not have at least one sample with >1 count per million (cpm) mapped reads or >64 total reads overall. 16 380 isotigs passed the filtering thresholds and were subject to further analyses. To assess differential expression, a 3 × 3 factorial design was used: three phenotypic groups (‘RR’, ‘WT’, and ‘IA’) and three dietary treatments [corn (0) or 8 or 36 h on soybean]. A 3 × 3 anova model was fit for the dataset using edgeR 3.0.8 (Robinson et al. 2010). For the overall anova test, raw *P*-values for each gene were adjusted using the false discovery rate method (controls the proportion of false positives among significant findings; FDR *P*-value < 0.2) (Storey and Tibshirani 2003). Detailed results from the analyses are included in Table S2. A principal components analysis (affycoretools 1.30.0) was then conducted on voom-transformed values (limma 3.14.4) (Smyth 2004) of the differentially expressed isotigs/contigs to examine the overall correlations between gene expression profiles and their phenotype/dietary treatments.

### Weighted gene correlation network analysis and GO enrichment analysis

Data including the isotigs/contigs that passed the significance threshold (FDR *P*-value < 0.2) were used to conduct Weighted gene correlation network analysis (WGCNA) (WGCNA 1.34 package; Appendix S1) (Zhang and Horvath 2005; Langfelder and Horvath 2008). The method allows grouping of isotigs/contigs into gene modules (with > 20 isotigs/contigs), such that all isotigs/contigs within the same module will have ‘similar’ expression patterns across the 18 Illumina sequencing samples; we used an unsigned analysis, such that isotigs/contigs that had large positive *or*negative correlations were placed in the same module (Figure S3A; Table S2). The expression pattern of all isotigs/contigs in one module can be summarized by calculating the first principal component score for each of the 18 samples, called ‘eigengene values’ that serve as indicators of the expression pattern within each module. The pattern of the eigengene values corresponds to the specific pattern shown by the majority of the isotigs/contigs in the module, but because of the unsigned analysis, other isotigs/contigs in the module will have the inverse to the eigengene pattern (Figure S3B). The eigengene values were subjected to an additional anova to identify modules that are responsive to the phenotypes or dietary treatments. For modules with significant ‘phenotype × diet interaction’, we compared the eigengene values among phenotypic groups within each separate dietary treatment using simple main effect tests (SPSS; IBM Inc., Chicago, IL, USA). Modules whose expressions were correlated with WCR phenotypes under at least one dietary treatment were subjected to GO enrichment analysis using the GOrilla software (significance threshold = 0.001; accessed May 2014) (Eden et al. 2009). For these analyses, GO terms were obtained from GOrilla using FlyBase identifiers. All isotigs/contigs that have orthologs in the *Drosophila*genome and passed the 1 cpm threshold were identified. Together, their best FlyBase (St Pierre et al. 2014) hits were included to form a ‘background set’, while those of isotigs/contigs within each module were used to form a separate ‘target set’. The complete lists of the enriched GO terms are shown in Tables S3 and S4. The results were then summarized using REViGO (cutoff value *C* = 0.5; including *P*-values) (Supek et al. 2011), which removes semantically redundant terms. For each module tested, enriched terms under the ‘biological process’ and ‘molecular function’ domain that had the top three lowest *P*-values were reported.

### Assessment of applied methods and analyses

Although the methods applied in this work have been previously adopted (Bonizzoni et al. 2011; Drnevich et al. 2012; Xue et al. 2013; Flagel et al. 2014), validation of our data was conducted using the expression profiles of a WCR cysteine protease gene *DvRS5*(Accession no.: KJ396941), which has been shown to be upregulated in RR-WCR adult guts relative to WT adults (Curzi et al. 2012). A search in our transcriptomic data revealed that *DvRS5*is a differentially expressed isotig that was grouped into a gene module transcriptionally responsive to the RR phenotype (Module F). *DvRS5*expression levels across WCR phenotypes determined by RNA-seq (Figure S4) and the eigengene value of Module F were congruent (Fig. 2); both of which also exhibited trends similar to that of a previous work on independent populations (i.e., expressed higher in RR-WCR, particularly on soybean diets) (Curzi et al. 2012). Thus, it is considered that the methods and analyses used in this work are suitable.

## Results

### Global transcription profiles in WT- and RR-WCR guts

**Figure 1:**
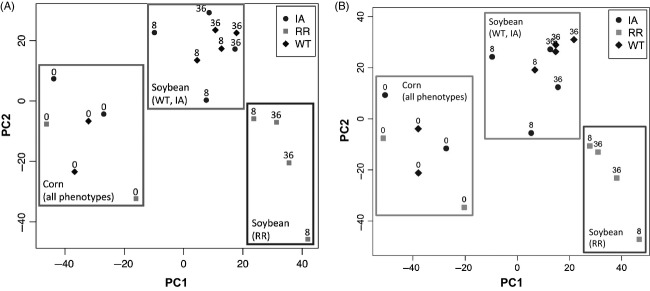
Principal component analysis on gene expression profiles in guts of different WCR populations fed on corn (designated as 0) or soybean foliage (8 or 36 h). Data included in the analysis: (A) 3973 differentially expressed isotigs/contigs and (B) all 16 380 isotigs/contigs. PC: principal component.

To determine whether the gut transcriptome of RR-WCR adults diverge from those of WT-WCR populations, we examined the gut transcription profiles of different WCR phenotypes fed on corn or soybean diets at a global scale. A two-way anova tests (phenotype × diet) on the Illumina data identified 3973 differentially expressed isotigs/contigs (of 16 380; overall test FDR < 0.2). A PCA on the expression data of these isotigs/contigs showed that gut transcriptomes of WT, IA, and RR population adults were relatively similar when fed on corn diets; however, the expression profiles of RR populations were different from those of WT and IA populations when WCR adults were fed on soybean foliage (Fig.1A). These patterns were similar to those from a PCA plot including data from all 16 380 isotigs/contigs (Fig.1B), indicating that the phenotype and dietary treatment indeed have strongest effects on the overall gene expression patterns.

### Gene modules correlated with the RR phenotype

**Figure 2:**
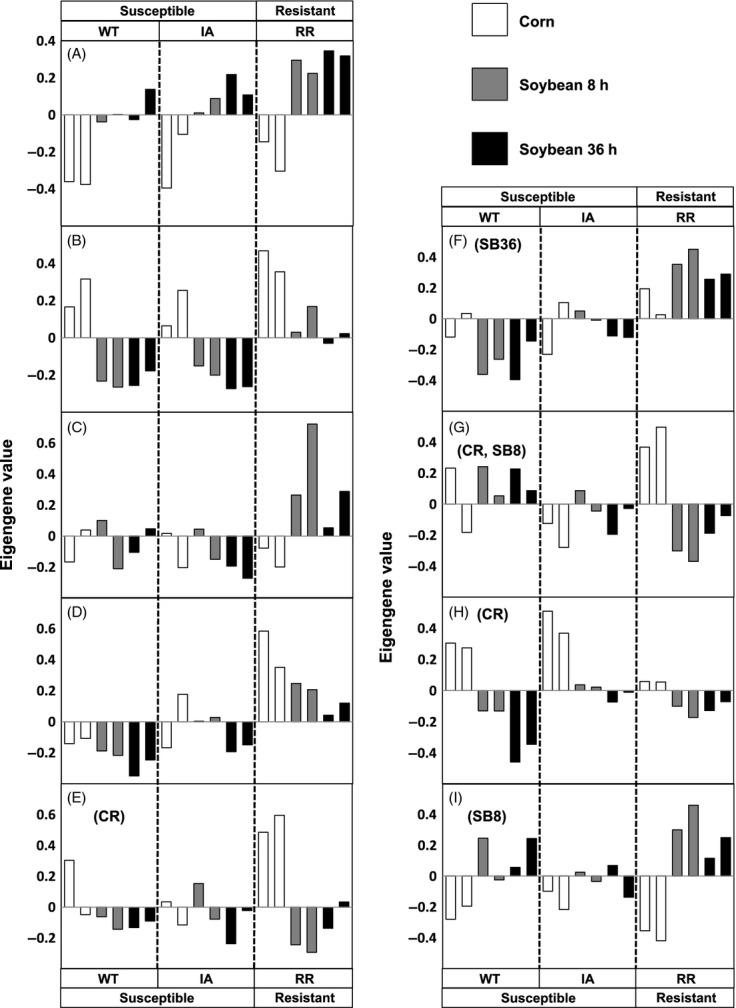
Expression patterns of modules whose expression is different between rotation-resistant (RR) and wild-type (WT and IA) WCR under at least one dietary condition. These data are shaded by the dietary treatments, while the phenotypic groups are indicated above/below the figures. The bar heights represent eigengene values of independent Illumina sequencing samples. Modules A–D did not have a significant ‘phenotype × diet’ interaction in a two-way anova test. The remaining modules had significant interactions and were subjected to simple main effect tests; the diets in which the eigengene values are correlated with the RR phenotype are labeled next to (or below) their module IDs. CR, corn; SB8, eight hours on soybean; SB36, thirty-six hours on soybean.

While the PCA clustering shows the strongest similarities and differences, individual isotigs/contigs may show other expression patterns across the phenotypes × diet groups. To assess these transcription patterns and understand what genes contributed to these patterns, we used WGCNA, statistical analyses of eigengene values, and GO enrichment analyses to identify/characterize gene modules with specific phenotype × diet patterns. We identified nine modules where the expression of RR is different from WT- and IA-WCR under at least one dietary treatment (Fig.2, Modules A to I), together representing 60.5% of the differentially expressed isotigs detected in this study. Modules A, B, and C exhibited similar expression profiles in WT and IA samples regardless of the dietary treatments, but demonstrated marked expression differences in RR samples. Genes in these modules are functionally related to metabolic processes (Modules A and B), transmembrane transport (Module A), immune responses (Module C), and exhibit catalytic or transporter activities (Module A), hydrolase (Module B), endopeptidase (Module C), or other activities (Table1). In Module D, the overall expression levels differed in all three phenotypic groups (Fisher’s LSD; *P* < 0.1). However, its expression levels in IA and WT samples were more similar compared to the RR samples.

**Table 1:** Highlights of results from GO enrichment analyses on gene modules potentially correlated with the RR phenotype.

Module #	Size*	Overall anova sig.†	Significant factor(s) influencing expression	Biological process‡		Molecular function‡	
				GO term ID	Description	GO term ID	Description
A	1078	4.5E-04	Phenotype, Diet	0055085	Transmembrane transport	0003824	Catalytic activity
				1901564	Organonitrogen compound metabolic process	0015145	Monosaccharide transmembrane transporter activity
				0005975	Carbohydrate metabolic process	1901476	Carbohydrate transporter activity
B	115	6.3E-05	Phenotype, Diet	1901071	Glucosamine-containing compound metabolic process	0008061	Chitin binding
				0006040	Amino sugar metabolic process	0016798	Hydrolase activity, acting on glycosyl bonds
				0006022	Aminoglycan metabolic process	0016787	Hydrolase activity
C	90	5.7E-02	Phenotype	0055114	Oxidation–reduction process	0004175	Endopeptidase activity
				0045087	Innate immune response	0004866	Endopeptidase inhibitor activity
				0008063	Toll signaling pathway	0016491	Oxidoreductase activity
D	59	1.0E-03	Phenotype, Diet	0009266	Response to temperature stimulus	ND	
				0055085	Transmembrane transport		
				0031427	Response to methotrexate		
E	466	5.7E-03	Diet, interaction	0051494	Negative regulation of cytoskeleton organization	0005515	Protein binding
				0040007	Growth	0008092	Cytoskeletal protein binding
				0051656	Establishment of organelle localization	0004871	Signal transducer activity[Table-fn tf1-5]
						0060089	Molecular transducer activity[Table-fn tf1-5]
F	243	2.8E-03	Phenotype, interaction	0007157	Heterophilic cell–cell adhesion	0015144	Carbohydrate transmembrane transporter activity
				0007155	Cell adhesion	1901476	Carbohydrate transporter activity
				0022610	Biological adhesion	0008236	Serine-type peptidase activity
G	110	8.6E-03	Phenotype, interaction	0009653	Anatomical structure morphogenesis	0005543	Phospholipid binding
				0034330	Cell junction organization	0050839	Cell adhesion molecule binding
				0045216	Cell–cell junction organization	0003779	Actin binding
H	138	2.0E-06	Phenotype, Diet, interaction	0042493	Response to drug	ND	
				0072347	Response to anesthetic		
I	104	1.7E-03	Diet, interaction	0005975	Carbohydrate metabolic process	0004867	Serine-type endopeptidase inhibitor activity

ND, No enriched GO terms detected.

* Number of isotigs/contigs included in each gene module.

† Significance values of overall anova tests on the eigengene values of each module; significance threshold: *P* < 0.1.

‡ Results from the gene list analyses were summarized with REViGO. For each GO domain (‘biological process’ or ‘molecular function’), three GO terms with the lowest *P*-values (from the summarized results) were reported. The significance threshold was set at 0.001 (default setting).

The two terms have the same *P*-value.

In Modules E, F, and G, the expression patterns were similar in WT and IA samples under at least one dietary treatment, yet both significantly differed from the RR eigengene values under the same diet (Bonferroni corrected; *P* < 0.1; Fig.2). Genes belonging to these modules were related to processes such as the regulation of cytoskeleton organization, growth (Module E), biological adhesion (Module F), anatomical structure morphogenesis, and cell junction organization (Module G; Table1). In Module H under the corn diet treatment, all three phenotypes had statistically different eigengene values, yet the eigengene values of IA and WT samples were more similar (Fig.2). In Module I under the soybean treatment (8 h), the expression trends were closer between IA and WT (*P* = 0.98) than between IA and RR (*P* = 0.02) or WT and RR (*P* = 0.12). These modules were related to an organism’s responses to drugs (Module H) and carbohydrate metabolic processes (Module I; Table1).

### Gene modules potentially correlated with phenotypic changes of WCR from Iowa

**Figure 3:**
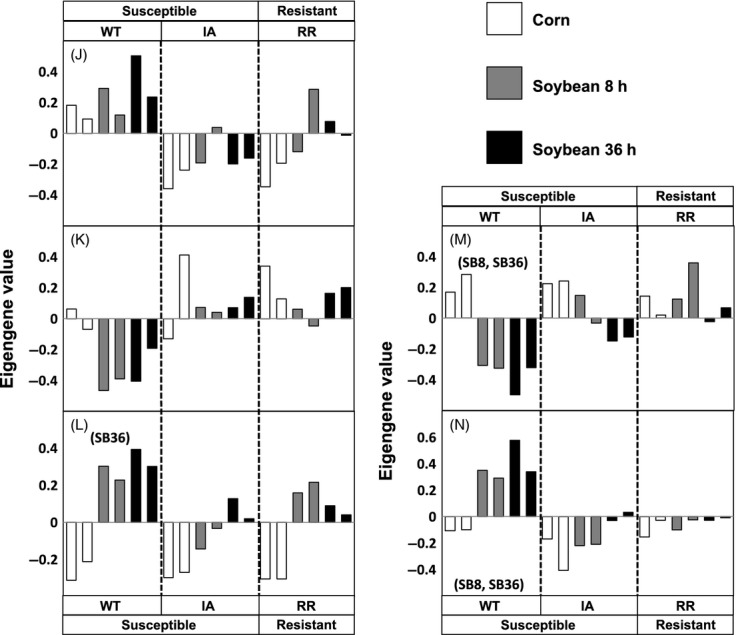
Expression patterns of modules whose expression is similar between rotation-resistant (RR) WCR and WCR from Ames, Iowa (yet different from other WT-WCR), under at least one dietary condition. These data are shaded by the dietary treatments, while the phenotypic groups are indicated above/below the figures. The bar heights represent eigengene values of the Illumina sequencing samples. Modules J and K did not have a significant ‘phenotype × diet’ interaction in a two-way anova test. The remaining modules had significant interactions and were subjected to simple main effect tests; the diets in which the eigengene values exhibit the pattern of interest are labeled next to (or below) their module IDs. CR, corn; SB8, eight hours on soybean; SB36, thirty-six hours on soybean.

A previous study found that WCR collected from Ames, Iowa, exhibited slightly higher tolerance of soybean than other WT populations examined (Curzi et al. 2012). Therefore, we searched for gene modules (with enriched biological process or function ontology terms) that may contribute to such a change. Five modules were identified showing similar expression patterns between IA and RR samples but differences in expression from WT samples. The isotigs/contigs included in these modules (Modules J to N; Fig.3 and Table2) represent 23.6% of the differentially expressed contigs/isotigs in our dataset. Modules J and K exhibited such patterns regardless of the dietary conditions, whereas the expression of Modules L, M, and N showed such patterns only when insects were fed on soybean diets. Genes belonging to these modules were related with various metabolic processes (Module J, K, and L), transmembrane transport (Module M), regulation of proteolysis (Module J), neuron homeostasis (Module N), and other biological processes/functions (Table2).

**Table 2:** Highlights of results from GO enrichment analyses on gene modules whose expressions are more similar between IA- and RR-WCR populations.

Module #	Size*	Overall anova sig.†	Significant factor(s)	Process‡		Function	
				GO term ID	Description	GO term ID	Description
J	69	1.4E-02	Phenotype, Diet	0045861	Negative regulation of proteolysis	0004867	Serine-type endopeptidase inhibitor activity
				0051346	Negative regulation of hydrolase activity	0080019	Fatty-acyl-CoA reductase (alcohol-forming) activity
				0043455	Regulation of secondary metabolic process	0004180	Carboxypeptidase activity
K	32	2.4E-02	Phenotype, Diet	0071616	Acyl-CoA biosynthetic process	0015645	Fatty acid ligase activity
				0035383	Thioester metabolic process	0016877	Ligase activity, forming carbon-sulfur bonds
L	682	4.0E-06	Phenotype, Diet, interaction	1901605	Alpha-amino acid metabolic process	0003824	Catalytic activity
				0006082	Organic acid metabolic process	0016742	Hydroxymethyl-, formyl- and related transferase activity
				0044281	Small molecule metabolic process	0019238	Cyclohydrolase activity
M	129	3.9E-04	Phenotype, Diet, interaction	0055085	Transmembrane transport	0046873	Metal ion transmembrane transporter activity
						0005215	Transporter activity
						0004553	Hydrolase activity, hydrolyzing O-glycosyl compounds
N	27	2.6E-04	Phenotype, Diet, interaction	1900073	Regulation of neuromuscular synaptic transmission	0000981	Sequence-specific DNA binding RNA polymerase II transcription factor activity
				0070050	Neuron homeostasis	0001077	RNA polymerase II core promoter proximal region sequence-specific DNA binding transcription factor activity involved in positive regulation of transcription
				0007254	JNK cascade	0043565	Sequence-specific DNA binding

ND, No enriched GO terms detected.

* Number of isotigs/contigs included in each gene module.

† Overall anova tests on the eigengene values of each module; significance threshold: *P* < 0.1.

‡ Results from the gene list analyses using GOrilla were summarized with REViGO by removing redundant GO terms. Three GO terms with the lowest *P*-values (from the summarized results) were reported for each gene module. The significance threshold was set at 0.001 (default setting).

## Discussion

Landscape changes associated with the annually rotated corn and soybean monoculture increased the WCR’s exposure to the nonhost soybean and have selected for beetles with altered behavior and gut physiology (Levine et al. 2002; Knolhoff et al. 2006; Pierce and Gray 2006; Gray et al. 2009; Curzi et al. 2012; Chu et al. 2013). Our study presents evidence suggesting that crop rotation has led to substantial changes in the gut transcriptome of the RR-WCR, particularly after exposure to soybean feeding (Fig.1). Many of the differentially expressed gene modules have potential physiological or immune functions that correspond to RR-WCR characteristics documented in previous studies (Curzi et al. 2012; Chu et al. 2013), which may enhance their fitness in agro-ecosystems characterized by the presence of a patchwork of corn and soybean fields, or even result in adaptive divergence among populations.

The global expression profiles of wild-type (‘WT’ and ‘IA’ samples) and rotation-resistant (‘RR’ samples) WCR adult guts were mostly similar when fed on corn, yet relatively different under soybean diets (Fig.1A). This pattern suggests that the regulation of a substantial portion of RR-WCR’s transcriptome is specifically responsive to brief soybean herbivory. A recent survey in eastern Iowa suggested that only low levels of RR existed in this area (Dunbar and Gassmann 2013). In the present study, the overall transcription patterns among phenotypes are in accordance with the suggested rare presence of the RR trait in Iowa (i.e., WT and IA have similar transcription profiles), yet also suggest that WCR populations in areas near the historical RR epicenter, Piper City, Illinois (Figure S1) (Levine and Oloumi-Sadeghi 1996), exhibit different genetic regulation that could be correlated with the RR phenotype.

The results of GO analyses indicate that gene modules transcriptionally correlated with the RR phenotype are related with a variety of physiological processes (Modules A to I; Fig.2; Table1), suggesting that regulation of various physiological aspects differs between RR- and WT-WCR. Within the nine responsive modules, Module A, B, and I were enriched with GO terms related with several metabolic processes and related functions (such as hydrolase, protease, and transporter activities). As previous findings have shown that gut physiology and tolerance of soybean diets differ between phenotypes (Curzi et al. 2012), it is possible to argue that metabolic pathways directly or subsequently correlated with the RR-WCR’s gut physiology could be transcriptionally adapted to optimize their overall performance under suboptimal dietary conditions. These physiological changes may also influence the regulation of their growth, morphology, or egg development in females – a possible explanation for the overrepresented terms identified in modules E and G, such as ‘growth’ or ‘anatomical structure morphogenesis’ (Table1). In addition, carbohydrate and energy reserve metabolism have been found to be correlated with insect behavioral patterns (Warburg and Yuval 1997). Nutritional homeostasis in insect hemolymph has also been suggested to correlate with locomotor activity and behavior (Simpson and Raubenheimer 1993; Lee and Park 2004). Thus, it is possible that transcriptional regulation of these modules may also be correlated with RR-WCR’s behavioral characteristics (e.g., elevated mobility).

Signaling and regulation of invertebrate immune responses are mediated by various proteases (Soderhall and Cerenius 1998; Kanost et al. 2004; Roh et al. 2009). In Module C, overrepresented GO terms related to immune responses and endopeptidase activities were identified under the ‘biological process’ and ‘molecular function’ domain, respectively (Table1). Animal gut microbiota compositions could be regulated by their host’s immune system (and vice versa) (Maynard et al. 2012; Broderick et al. 2014). As host–microbe interactions of RR-WCR differ from those of WT-WCR populations (Chu et al. 2013), it is possible that their immune genes are regulated differently. Thus, there may be an association between Module C’s regulation and the host–microbe associations in WCR (Chu et al. 2013). In Module F, we found that isotigs functionally related with biological adhesion (attachment of a cell or organism to other substrates or organisms) were also differentially transcribed between phenotypes. It has been shown that biological adhesion could function in invertebrate immune responses (e.g., phagocytosis and encapsulation) (Foukas et al. 1998; Holmblad and Söderhäll 1999). Other studies also showed that proteins related to such processes could contribute to the intestinal stem cell and progenitor cell biology of insects (Maeda et al. 2008; Marthiens et al. 2010) – processes that could be influenced by interaction with the gut microbiota or exposure to stress (Buchon et al. 2009; Chatterjee and Ip 2009). Therefore, the genes included in this module may also be related with host–microbe associations in RR-WCR. Other than such interactions, the possibility that immune responses and immune gene expression also correlate to other aspects of RR-WCR biology cannot be rejected. Immune-challenged caterpillars (*Grammia incorrupta*) have been shown to exhibit different feeding behaviors compared to untreated insects (Mason et al. 2014). Differential expression of genes associated with immune responses has also been found between *Drosophila*lines selected for different locomotor reactivity (Jordan et al. 2007). Therefore, it is possible that an altered immune system may be influencing WCR’s behavior and mobility as well.

Although a previous study found that WCR collected from Ames, Iowa, exhibited slightly higher tolerance of soybean than other WT populations (Curzi et al. 2012), here, the global gene expression profiles were relatively similar between WT-WCR from Ames, Iowa and the other WT-WCR when under different diets. This suggests that genetic regulation in their guts was similar despite the slightly elevated tolerance of soybean found in the former (Fig.1). Nevertheless, we also identified gene modules exhibiting similar expression patterns between RR populations and WCR from Ames, Iowa, albeit fewer and smaller in size. Most of such modules (Modules J, K, L, and N) were either related with metabolic processes or regulation of the nervous system (Table2), which may correspond to the phenotypic traits found in IA- and RR-WCR populations. Overall, although the roles of the identified gene modules in RR-WCR’s adaptation to crop rotation deserve further investigation, our results could help elucidate links between RR-WCR genetic regulation and their distinctive physiology.

Characterization of genetic bases for rotation-resistance and phenotypic traits of RR populations has been a formidable task due to the lack of reliable genetically diagnostic markers, and the complex behavioral, physiological, and morphological changes involved (Levine et al. 2002; Miller et al. 2006, 2007; Pierce and Gray 2006; Curzi et al. 2012; Mikac et al. 2013). Transcriptomic analyses focusing on RR-WCR guts provided insights into their physiological adaptation to brief soybean herbivory that would have been overlooked or not readily uncovered via traditional approaches. Our previous studies suggested that elevated digestive proteases activity and changes in host–microbe interactions provide RR-WCR tolerance of brief soybean herbivory (Curzi et al. 2012; Chu et al. 2013). Results from our present study suggest that multiple genetic regulations that are potentially linked with such traits or other related/unexplored physiological characteristics may also differ between RR- and WT-WCR (Table1).

It has been proposed that divergent selection on a trait can also influence other correlated traits (West-Eberhard 1989; Nosil 2012). If variation in genetic regulation or alleles that benefit the stabilization of the RR trait (or improve the fitness of individuals exhibiting RR) exists in the field, it is possible that some individuals may be favored by selection in resistant populations, given that the selection pressure (imposed by crop rotation) is maintained. Selection for RR may have led to divergence in other aspects of physiology or phenotypic plasticity, which could be reflected in their transcriptome. Whether these changes directly contribute to the RR-WCR’s altered behavior or are pleiotropic effects or subsequent adaptations following the reduced ovipositional fidelity to corn deserves further investigation. Nevertheless, the transcription patterns shown in this study could provide candidate targets for characterization of genetic mechanisms that underlie RR-WCR phenotypic traits and could be applied to develop methods for detecting RR individuals in the field. From a broader perspective, our findings suggest that complex changes in insect genetics/physiology could accompany the evolution of resistance to pest control measures. These data provide a more holistic view of how insect pests adapt to agricultural practices and suggest that such systems may serve as good subjects for investigating organismal variation, phenotypic plasticity, and evolution.

## Data archiving statement

The sequencing data and other information have been deposited in the Gene Expression Omnibus (GEO) database (accession number: GSE62480).

## Supporting Information

**Figure S1**. Adult WCR sampling sites.

**Figure S2**. Flow diagram describing strategies used to resolve the WCR adult gut transcriptome isogroups (i.e., genes) to single best representative isotigs (i.e., mRNA).

**Figure S3**. The relationship between the actual patterns of the isotigs/contigs in Module A versus the eigengene values.

**Figure S4**. Congruence between (A) averaged expression levels of cysteine protease gene *DvRS5*across different WCR phenotypic groups × dietary treatments (determined by RNA-seq; counts per million) and (B) the expression patterns of Gene Module F (averaged eigengene values).

**Table S1**. Sampling locations, characteristics, and usage of western corn rootworm populations included in this work.

**Table S2**. Details of results of the Illumina sequencing analyses.

**Table S3**. Over-represented biological process ontology terms of gene modules described in this study.

**Table S4**. Over-represented molecular function ontology terms of gene modules described in this study.
