# Supplementary material for: Patterns of differential gene expression in adult rotation-resistant and wild-type western corn rootworm digestive tracts
Source: Evol Appl. 2015 Jul 16;8(7):692–704. doi: 10.1111/eva.12278 (PMC4516421; doi:10.1111/eva.12278)
Supplement: Supplementary file 1 [file eva0008-0692-sd1.pdf]

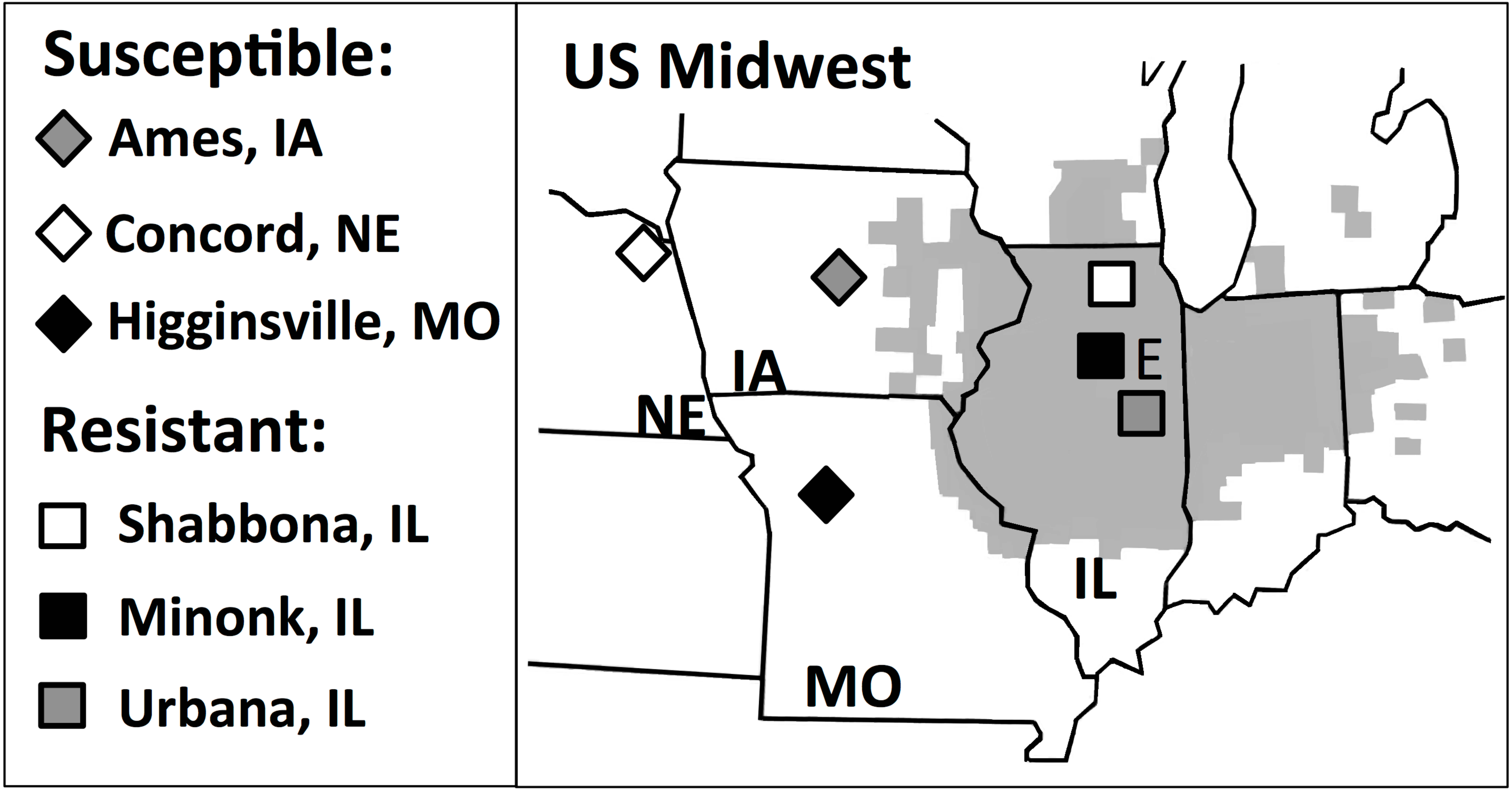
Susceptible:
Ames, IA
Concord, NE
Higginsville, MO
Resistant:
Shabbona, IL
Minonk, IL
Urbana, IL
US Midwest
IA
NE
MO
IL
E
