# Supplementary figures and images for: Patterns of differential gene expression in adult rotation-resistant and wild-type western corn rootworm digestive tracts
Source: Evol Appl. 2015 Jul 16;8(7):692–704. doi: 10.1111/eva.12278 (PMC4516421; doi:10.1111/eva.12278)

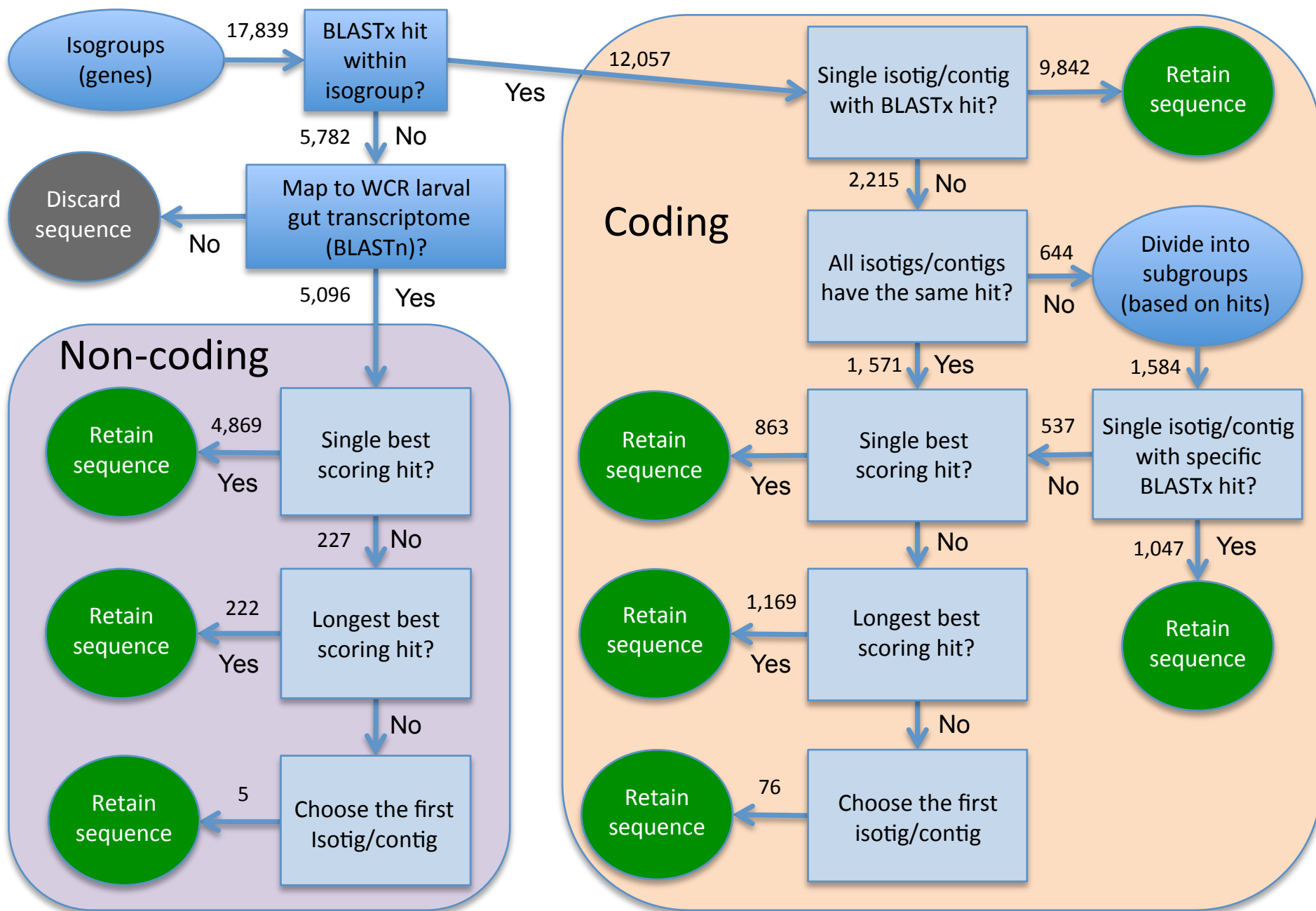

Supplement: Supplementary file 2 [file eva0008-0692-sd2.pdf]

A

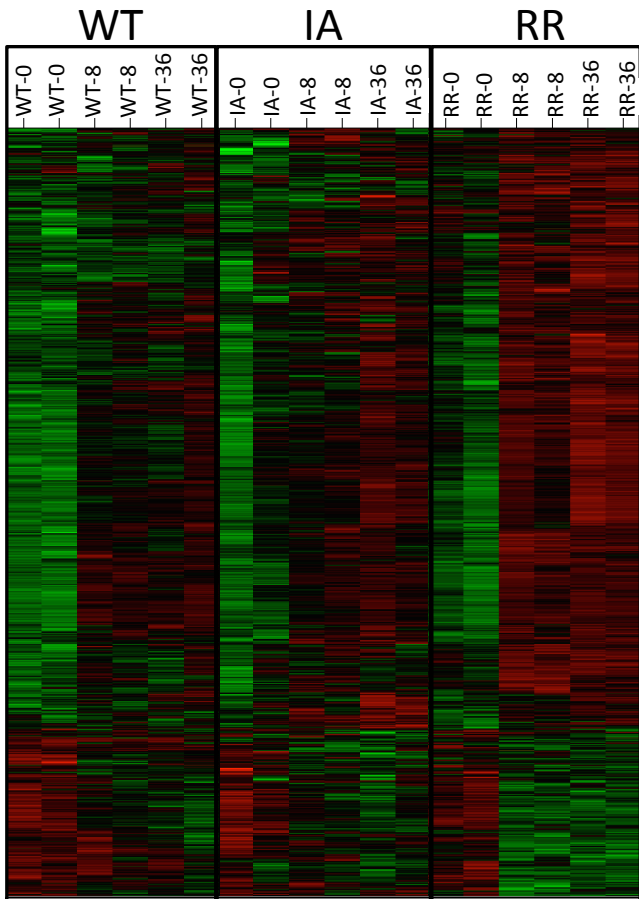

B

Eigengene value

0.4  
0.2  
0  
-0.2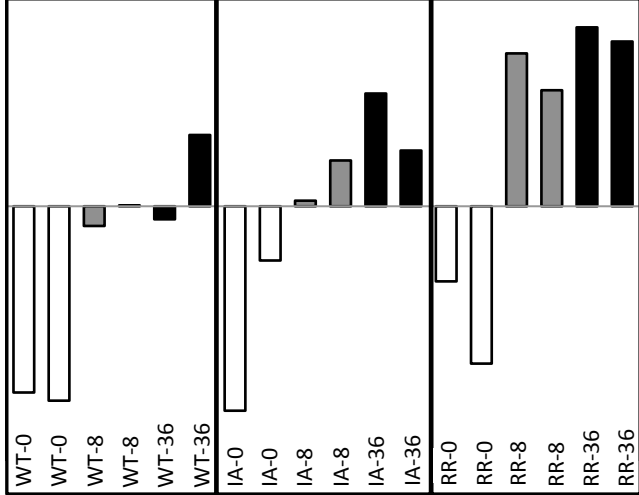

Supplement: Supplementary file 3 [file eva0008-0692-sd3.pdf]

**A**      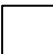 **Corn**      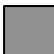 **Soybean 8h**      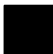 **Soybean 36h**

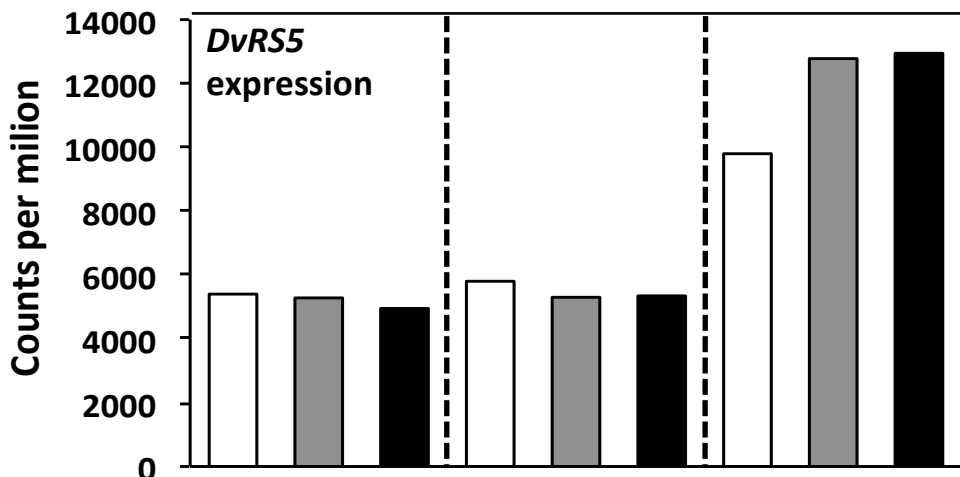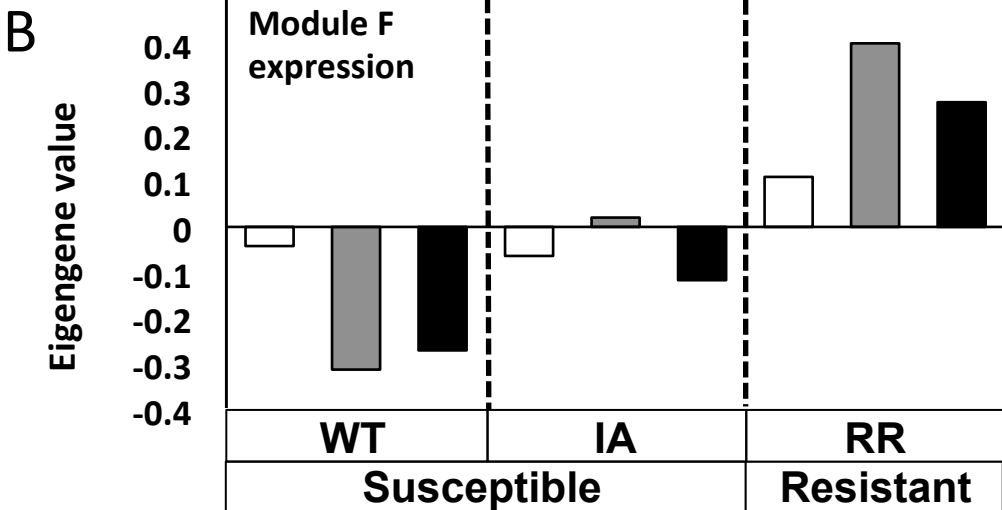

Supplement: Supplementary file 4 [file eva0008-0692-sd4.pdf]
