# Supplementary material for: Patterns of differential gene expression in adult rotation-resistant and wild-type western corn rootworm digestive tracts
Source: Evol Appl. 2015 Jul 16;8(7):692–704. doi: 10.1111/eva.12278 (PMC4516421; doi:10.1111/eva.12278)
Supplement: Supplementary file 5 [file eva0008-0692-sd5.docx]

| Table S1. Sampling locations, characteristics, and usage of western corn rootworm populations included in this work. | | | |
| --- | --- | --- | --- |
| Location | Field  coordinate | Resistance status /description | Usage |
| Brookings, SD | N/A | WT (non-diapausing lab colony) | Reference transcriptome |
| Concord, NE | 42°23'39''N, 96°57'23''W | WT | Reference transcriptome,  RNA-seq analysis |
| Higginsville, MO | 39°07'09''N 93°49'42''W | WT | Reference transcriptome,  RNA-seq analysis |
| Ames, IA | 42°3'8''N, 93°32'6''W | WT (exhibit slightly higher tolerance of soybean diet than the other WT populations) | Reference transcriptome,  RNA-seq analysis |
| Shabbona, IL | 41°50'36''N, 88°50'58''W | RR | Reference transcriptome,  RNA-seq analysis |
| Minonk, IL | 40°51'26''N, 89°00'26''W | RR | Reference transcriptome,  RNA-seq analysis |
| Urbana, IL | 40°09'14''N, 88°08'40''W | RR | Reference transcriptome,  RNA-seq analysis |

N/A: not applicable; WT: wild-type; RR: rotation-resistant.
