# Supplementary material for: Patterns of differential gene expression in adult rotation-resistant and wild-type western corn rootworm digestive tracts
Source: Evol Appl. 2015 Jul 16;8(7):692–704. doi: 10.1111/eva.12278 (PMC4516421; doi:10.1111/eva.12278)
Supplement: Supplementary file 9 [file eva0008-0692-sd9.docx]

**Methods**

**Sequencing procedures.** Both 454 and Illumina sequencing were conducted at the W.M. Keck Center for Comparative and Functional Genomics, Roy J. Carver Biotechnology Center, University of Illinois at Urbana-Champaign. Prior to sequencing, sample qualities were examined using the Agilent 2100 bioanalyzer (Agilent Technologies Inc., Santa Clara, CA). For 454 sequencing, mRNA was obtained from 21 micrograms of WCR gut RNA using the Oligotex kit (Qiagen Inc., Valencia, CA). The mRNA-enriched fraction was processed into a normalized cDNA library (with 454-compatible adaptors) as previously described (Lambert et al. 2010). The constructed library was tested using the Invitrogen Qubit fluorometer. The average fragment sizes were also measured by analyzing the library (1 microliter) with an Agilent DNA 7500 chip and a Bioanalyzer. The library was then diluted (1x10^6^ molecules per microliter). Afterwards, emulsion-based clonal amplification (emPCR) and sequencing (full plate) were conducted on the 454 Genome Sequencer FLX+ system (454 Life Sciences, Branford, CT). The bundled 454 Data Analysis Software v2.6 was used for signal processing and base calling. For Illumina sequencing, RNA-seq libraries were prepared with Illumina's TruSeq RNA Sample Prep kit. Both single and paired-end sequencing were used in this study. For single-end reads, the libraries were quantitated by qPCR, and sequenced on two lanes for 101 cycles on an Illumina HiSeq2000 (Illumina Inc., San Diego, CA) using V3 SBS sequencing chemistry. For paired-end reads, the same libraries were sequenced on one lane for 101 cycles from each end. FASTQ files were generated and demultiplexed using Casava 1.8.2 (Illumina Inc., San Diego, CA).

**Weighted gene correlation network analysis (WGCNA).** The WGCNA analysis (using the “blockwiseModules” function) begins by calculating Pearson correlation values between all possible pairs of genes. The following analysis was “unsigned” in that isotigs with strong positive/negative correlations were grouped in the same module. The correlation values were then transformed into an adjacency matrix using a power function, β (in this case the value was 9). This matrix was then used to calculate a topological overlap matrix (Zhao et al. 2010). After the values were subtracted from 1, the resulting distance matrix was then subjected to a hierarchical clustering analysis. Modules (including at least 20 isotigs) were then determined based on the clustering and merging of the isotigs (deepSplit = 2; mergeCutHeight = 0.2).

**References**

Lambert, J. D., X. Y. Chan, B. Spiecker, and H. C. Sweet. 2010. Characterizing the embryonic transcriptome of the snail *Ilyanassa*. Integrative and Comparative Biology **50**:768-77.

Zhao, W., P. Langfelder, T. Fuller, J. Dong, A. Li, and S. Hovarth. 2010. Weighted gene coexpression network analysis: state of the art. Journal of Biopharmaceutical Statistics **20**:281-300.
